# Emulsification Characteristics Using a Dynamic Woven Metal Microscreen Membrane

**DOI:** 10.3390/membranes6020034

**Published:** 2016-06-20

**Authors:** Rana Sabouni, Hassan G. Gomaa, Jiangshan Liu, Jesse Zhu

**Affiliations:** 1 Chemical and Biochemical Engineering Department, Western University, London, ON N6A 5B9, Canada; rasabouni@uwo.ca (R.S.); liujiangshan916@gmail.com (J.L.); jzhu@uwo.ca (J.Z.); 2 Department of Chemical Engineering, American University of Sharjah, Sharjah, United Arab Emirates

**Keywords:** membrane, emulsification, oscillatory motion

## Abstract

An oscillatory emulsification system for the production of oil in water emulsions using a commercially available low-cost woven metal microscreen (WMMS) is investigated. The system allows for independent control of both the oscillation frequencies and amplitudes such that it provides two degrees of freedom for controlling the emulsion properties. The investigations included the production of both surfactant and particle-stabilized emulsions. The average droplet size was found to decrease when both the oscillation frequency and amplitude was increased. For surfactant-stabilized emulsions, using bi-surfactants in both the continuous and dispersed phases resulted in a smaller droplet size due to lower interfacial tension. For particle-stabilized emulsions, both the hydrodynamics of the system and the hydrophobic and hydrophilic nature of the stabilizing particles influenced the interfacial properties at the oil–water interface, which in turn affected the final droplet size and distribution with potential droplet breakage. In absence of the latter, a simple torque balance model can be used to reasonably predict the average emulsion droplet size.

## 1. Introduction

Emulsification is an important unit operation in many industries such as pharmaceuticals, food, cosmetics, and fuel applications. It involves dispersing one immiscible liquid into another using equipment such as high-pressure homogenization or colloid mills. These high shear systems typically require high energy that partially dissipates in the form of heat with potential negative impact on product functional properties and the process energy efficiency. Among the approaches proposed to overcome such limitations is emulsification using membranes or microsieves [1,2]. In this technique, pressure is applied to force one phase to permeate into another to produce an emulsion. Droplet detachment in this case is basically induced when the shear force exerted by the continuous phase exceeds the capillary force holding the droplets to the membrane pores.

To further enhance the technique ability to produce high concentration dispersed phase without recycling, which can damage the formed droplets, dynamic membrane emulsification was proposed to decouple the continuous phase flow from surface shear by adding another degree of freedom using for example stirrer [3,4] or surface rotation [5,6,7,8]. Oscillatory motion has also been investigated for membrane emulsification since it provides a potential for better control of shear and consequently particle size by allowing two degrees of freedom using either oscillation frequency and/or amplitude. Furthermore, and as shown in dynamic membrane filtration, the energy density in a well-designed oscillatory system can be less than that in other dynamic membrane systems [9]. Among these investigations is the use of a micro-machined membranes excited by a piezo actuator system at very small amplitudes (~10–12 µm), where a decrease in droplet size was reported at frequencies of ~10 Hz [10]. Both fouling and inertia of the experimental setup, however, prevented the obtainment of results at higher excitation forces. In another investigation, a comparison was made between emulsions produced using tubular needle oscillating membrane to that of a rotating disc on top of stationary membrane. The results showed that using oscillations gave smaller and more uniform drops and that drop size decreased with increasing the oscillatory surface shear [11]. This agreed with the numerical simulations of the effect of the vibration on the droplet formation at the single pore, thus confirming the effect of surface shear on enhancing droplet detachment [12]. The effectiveness of oscillatory motion in reducing and controlling droplet size in membrane emulsification has also been demonstrated recently using an azimuthally oscillating membrane [13], and by imposing an oscillatory flow component on crossflow in membrane emulsification [14].

In most of the previous investigations, either specially designed membranes or micro-machined sieves were used, which in some cases suffered from poor mechanical strength, low porosity, brittleness, high cost, or a combination of these. Furthermore, the range of oscillation frequencies and amplitudes in some designs could not be independently changed. This contribution presents oil in water emulsification results in an oscillatory membrane system using a commercially available woven metal microscreen (WMMS) that is oscillated at frequencies and amplitudes that can be controlled independently. WMMS’s have been used in many applications including solar-receiving devices, catalytic reactors, and fluid filtration due to their good chemical and thermal properties as well as their superior mechanical strength in comparison to other non-metal materials [15,16]. Different emulsification conditions are presented including both surfactant-stabilized as well as particle-stabilized emulsions. The latter, also known as Pickering emulsions (PE), has recently gained significant interest due to their higher stability and resistance to coalescence. Furthermore, using benign solid particles instead of surfactants can provide additional functionality and possibly avoid the adverse effects that may be linked to surfactants in cosmetics and pharmaceutical applications [17,18]. The variation of the emulsion average droplet size and distribution over a wide range of oscillation frequencies and amplitudes, as well as dispersed phase flux are herein discussed. A comparison of the measured average droplet size with theoretical predictions is also reported.

## 2. Materials and Methods

Commercial grade vegetable oil was used as the dispersed phase, while the continuous phase was demineralized water containing an emulsion-stabilizing agent. The experimental setup consisted of a rectangular container filled with the continuous phase and two stainless steel woven metal microscreens (WMMS’s) with a 38-µm pore size and 36% porosity housed on both sides of a flat surface frame that oscillated vertically using an adjustable eccentric to provide a wide range of oscillatory frequencies and amplitudes (*f* = 0–25 Hz and *a* = 0–22 mm). The walls of the container were equipped with horizontal, 5-mm stainless steel flat strips that acted as mild turbulence promoters due to the interaction with the secondary flow formed by fluid displacement as the unit oscillated without obstructing the emulsification area. The dispersed phase was injected through the microscreen by a peristaltic pump with a variable speed motor to control the flow rate (Pharmacia Fine Chemicals P-3 Peristaltic Pump, Piscataway, NJ, USA). Before each experiment, the module was cleaned and rinsed using ethanol and demineralized water to eliminate any cross contamination. All experiments were conducted at a room temperature of 22 ± 0.5 °C. The interfacial tensions were measured using an FTA1000 Drop Shape Instrument, B Frame System (First Ten Angstrom, Portsmouth, VA, USA), with FTA video drop shape software by First Ten Angstroms (Portsmouth, VA, USA). The viscosity was measured using a Brookfield Engineering Laboratories rheometer model LVDVE115 (Brookfield AMETEK, Middleboro, MA, USA).

### 2.1. Emulsion Stabilization

#### 2.1.1. Surfactant-Stabilized Emulsion

Water-soluble emulsifier Tween-20 (polyoxyethylene sorbitan monolaurate-2%) was used in the continuous phase. For the bi-emulsifier system, 4% Span-80 (sorbitan monooleate) was used in the dispersed phase to ensure fast availability of emulsifier molecules at the forming interfaces to minimize coalescence between adjacent pores.

#### 2.1.2. Pickering Emulsions (PE)

The stabilizing particles used included hydrophilic fumed silica, 4A- zeolite, and metal organic frameworks (MOFs), namely, ZIF-8 and MIL-101. All materials were purchased from Sigma-Aldrich except the MOFs. ZIF-8 was synthesized by dissolving a solution of Zn(NO_3_)_2_·6H_2_O in methanol followed by adding 2-methylimidazol and stirring for 1 h. The white precipitate was separated by centrifugation and dried in a vacuum oven (120 °C) for 24 h. MIL-101 MOFs was prepared by dissolving mixture of NH_2_-terphthalic acid and of FeCl_3_ in dimethylformamide followed by microwave thermal treatment for 30–90 s. The product was filtered, washed, and then dried in a vacuum oven at 25 °C [19]. Before each experiment, the particles were stirred in distilled water for 15 min and then mixed with the continuous phase to ensure homogenous distribution.

### 2.2. Emulsion Preparation and Characterization

All emulsions were prepared by injecting 12.5 mL of oil (dispersed phase) in 400 mL of the continuous phase. Samples were collected at the end of each experiment for emulsion characterization using digital microscopy (Zeiss M2 1256 microscope, Oberkochen, Germany). The average of triplicate measurements was used to determine the droplet size and distribution. The images of approximately 100–150 droplets in the selected field of view were analyzed both manually and using image analysis software (ImagePro, Rockville, MD, USA) and ImageJ, (Bethesda, MD, USA). The droplet size distribution was expressed using the coefficient of variation (CV) for *n* droplets using

(1) CV=1d∑in[(di−d)2n]12

**Figure 1:**
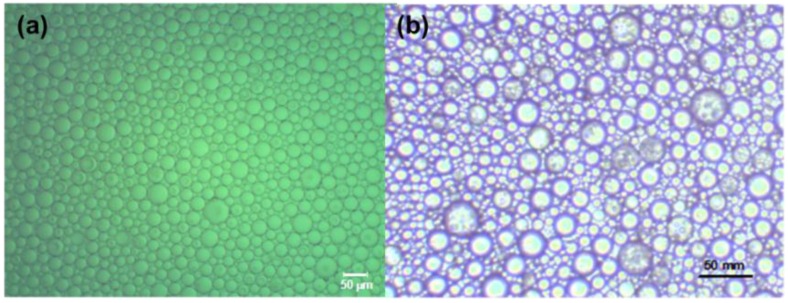
Micrograph of emulsions stabilized with (**a**) surfactant (*f* = 10 Hz, *a* = 6 mm, *J* = 50 LMH); and (**b**) metal organic frameworks (MOF) particles (*f* = 18 Hz, *a* = 20 mm, *J* = 72 LMH). LMH: liter per meter square per hour.

Figure 1 shows representation for typical emulsion droplets produced using both surfactants and particles for emulsion stabilization, which shows good droplet size uniformity.

## 3. Results and Discussion

### 3.1. The Effect of Oscillation

Droplet detachment in membrane emulsification occurs mainly when the shear caused by the drag force created by the relative motion between the fluid and the surface exceeds the interfacial tension holding force, which keeps the droplet attached to the surface pore. The size of the droplet at detachment time will then be determined by the balance between both forces. As the droplet continues to grow, its height increases, resulting in a higher drag force acting on its surface until such force overcomes the capillary holding force resulting in droplet detachment. For a membrane oscillating harmonically in a fluid with kinematic viscosity *v_c_* and density *ρ_c_* with amplitude *a* and angular frequency ω, the maximum shear stress τ at the fluid-surface interface increases with increasing the oscillation amplitude or frequency as [20]

(2) $$\tau =a\omega^{3/2}\left(\rho_{c}\nu_{c}^{1/2}\right)$$

Accordingly, increasing the oscillation intensity would be expected to result in smaller droplet size since the match point between the shear and the capillary forces will be reached at an earlier time. Oscillations can also affect the droplet size through its potential influence on the interfacial tension, as well as the particle stability in particle-stabilized emulsions, as will be discussed later.

#### 3.1.1. Surfactant-Stabilized Emulsions

**Figure 2:**
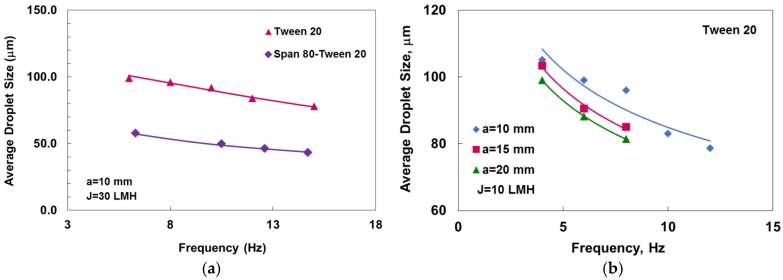
Change of droplet size with oscillation frequency. (**a**) Effect of surfactants; (**b**) effect of amplitude.

As indicated before, increasing the oscillation intensity either by increasing the frequency or the amplitude is expected to decrease the emulsion average droplet diameters, which can be clearly seen in Figure 2. Furthermore, two main other observations can be seen from the graph. First is the relatively smaller droplet size, which is typically between two to ten times larger than the pore size. The second observation is the narrow CV values, which reflect a good size distribution.

Using Span 80–Tween-20 bi-emulsifier resulted in a smaller average droplet size than that obtained when using Tween-20 alone. This can be expected since the interfacial tension of the bi-surfactant is lower, which decreases the droplet-pore holding force, promotes earlier detachment, and minimizes the potential for droplet coalescence. The presence of surfactant in the dispersed phase also allows for faster action at the droplet surface interface due to the faster diffusion time to the droplet interface compared with a surfactant in continuous phase. This can be related to the fact that, in membrane emulsification, as the droplets form at the membrane pores, the surfactant concentration in the continuous phase becomes depleted near the droplets surface. If the diffusion rate of the surfactant molecules is fast enough to keep up with the droplet surface growth, it will continue to provide the necessary surface coverage to maintain low interfacial tension. The opposite will occur if the surfactant diffusion rate is slow compared with its depletion rate, which is more likely to occur in the continuous phase since it will have to diffuse through the boundary layer to reach the droplet surface.

**Figure 3:**
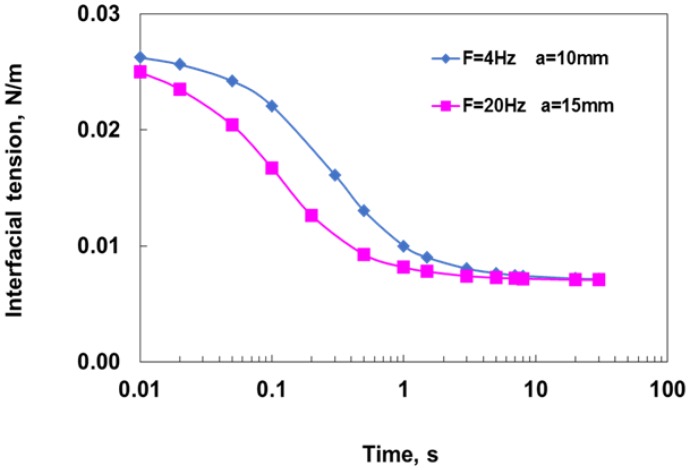
Calculated change in dynamic interfacial tension with oscillations determined from the solution of Equation (3).

The change in the dynamic interfacial tension γ(*t*) with time can be related to the pure surface interfacial tension γ*_o_* and the dynamic surface adsorption Γ(*t*) using the Langmuir isotherm:

(3) $$\gamma \left(t\right)=\gamma_{o}+nRT\times \Gamma_{\infty }\times \ln\left(1-\frac{\Gamma \left(t\right)}{\Gamma_{\infty }}\right)$$

where *n* = 1 for neutral molecules, *n* = 2 for ionic, Γ∞ is the limiting equilibrium surface accumulation, *R* the universal gas constant, and T the absolute temperature. If Cb and Cs are the surfactant bulk and surface concentrations, respectively, then the mass transfer equations can be written in the following form:

(4) $$\frac{d\Gamma \left(t\right)}{dt}+\alpha \Gamma \left(t\right)=k_{c}\left(C_{b}-C_{s}\right)$$

in which α is the droplet surface area expansion rate, which for generation time *t* is α = 2/3*t* [21,22], and *k_c_* is the mass transfer coefficient. Integration of the above equations gives the change in the interfacial tension with time. Since the diffusion rate scales with the mass transfer coefficient increases as the oscillation intensities increase [23], it would be expected that higher oscillations would result in faster surfactant diffusion rate and surface coverage during droplet formation, consequently facilitating the formation of a smaller droplet size due to the lower capillary force and coalescence potential. Figure 3 shows the calculated dynamic interfacial tension for different oscillation conditions, which, as expected, decreases with increasing oscillations due to the associated increase in the surfactant transfer rate.

**Figure 4:**
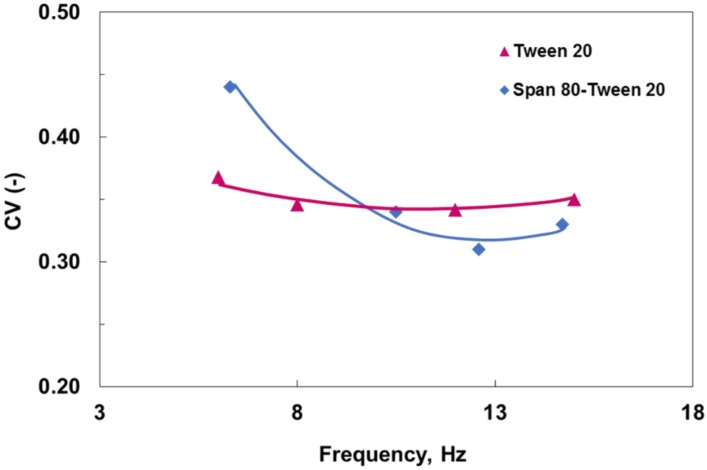
Change in coefficient of variation (CV) with oscillation frequency for surfactant-stabilized emulsions. Dispersed phase flux *J* = 30 L·m^−2^·h^−1^ (LMH), oscillation amplitude, *a* = 10 mm.

Figure 4 shows the effect of oscillation and surfactants on droplet size distribution. As can be seen, the latter decreased as the frequency increased, and the effect was much stronger initially for the case of the bi-surfactant systems, indicating a narrower drop size distribution. Such a trend, however, did not continue, as CV values reached a minimum and then increased again as the frequency was increased.

**Figure 5:**
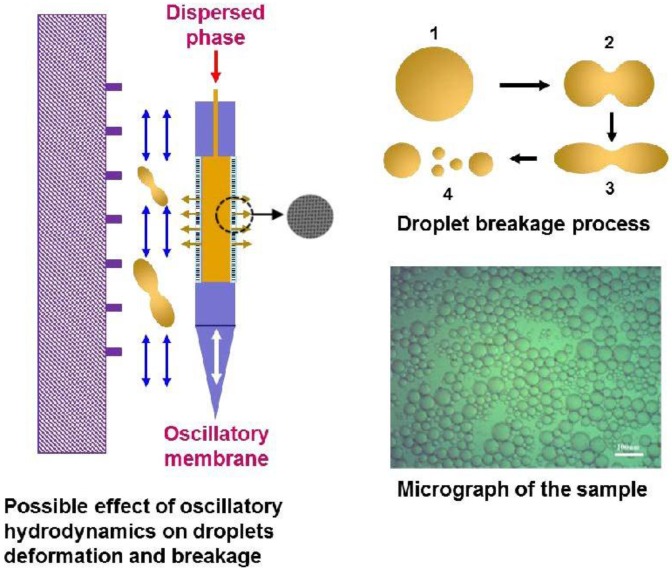
Schematic illustration of a proposed mechanism of droplet breakage at high oscillations. Micrograph is for dispersed phase flux *J* = 30 LMH, oscillation frequency = 14.8 Hz, amplitude = 10 mm.

A possible explanation for such behavior could be related to the droplet deformation and breakage into smaller satellite ones as illustrated in Figure 5. Such phenomena are more likely to occur when larger droplets are formed at low oscillation intensities with a greater tendency toward deformation and breakage. Similar behavior may also occur at high oscillation intensities, particularly if the interfacial tension is low, which promotes the formation of smaller droplets. This may provide some explanation to the observed increase in CV values at higher frequencies for the bi-surfactant system in comparison to those for the single surfactant.

#### 3.1.2. Particle-Stabilized Emulsions

**Figure 6:**
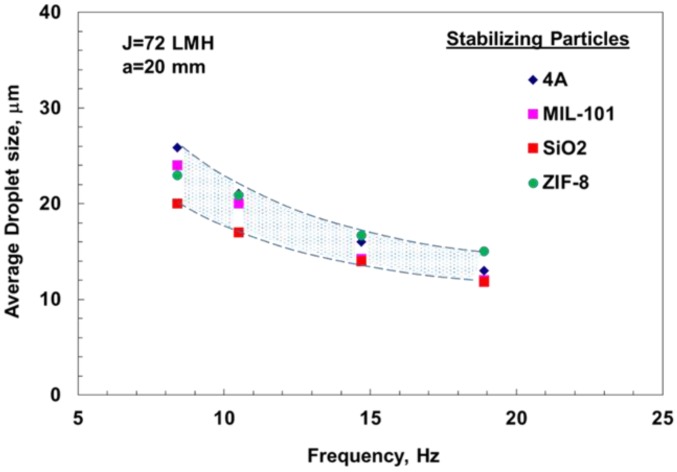
Change in droplet size with frequency for different particle-stabilized emulsions. Measurements were made at dispersed phase flux *J* = 72 L·m^−2^·h^−1^ (LMH).

The stabilization mechanism of Pickering emulsions is mainly based on the adsorption of solid-particles at the oil–water interface where the particle becomes trapped in deep energy that well exceeds the Brownian thermal energy for the system, thus creating a physical barrier that prevents droplet coalescence [24]. The stabilization can also be contributed in some cases to interfacial tension reduction similar to classical surfactants [25]. The thermodynamic state for the latter in absence of particles interactions is

(5) $$\Delta \gamma =NE_{d}/A$$

in which Δγ is the change in the system interfacial tension due to the presence of particles, *N_p_* is the number of particles in a given interfacial area *A*, and *E_d_*, is the desorption energy required to remove one particle from the interface given by,

(6) $$E_{d}=\pi R_{p}^{2}\gamma_{o}{\left(1-\left|\cos\theta \right|\right)}^{2}$$

in which *R_p_* is the particle radius, γ*_o_* is the interfacial tension of the oil–water nude interface, and θ is the particle contact angle with the oil–water interface. For particle stabilized emulsions, and assuming particle adsorption is thermodynamically favored, the kinetics for particle barrier formation must be matched to the drop-by-drop formation process to provide sufficient stability at the membrane surface to prevent coalescence. This means the particles need to be at the surface long enough in order to preferentially wet the interface and then remain irreversibly adsorbed, which strongly depends on the particle affinity to the surface. If the particle is weakly attached to the interface, it could diffuse away or become stripped off, depending on the system characteristics and hydrodynamics. Accordingly, it would be expected that strongly hydrophobic particles will have a greater affinity toward the oil droplet and stronger attachment force that can resist both thermal and hydrodynamic detachment forces and will in turn result in emulsions with a stable droplet size. The experimental results depicted in Figure 6 show a general declining trend in the average droplet size of particle-stabilized emulsions as the oscillation intensity for different stabilizing particles increases, similar to surfactant-stabilized emulsions. Furthermore, it can be seen that it was possible to produce droplets with sizes smaller than that of the pore size, which, as indicated earlier, is typically orders of magnitude larger.

### 3.2. The Effect of Dispersed Phase Flux

**Figure 7:**
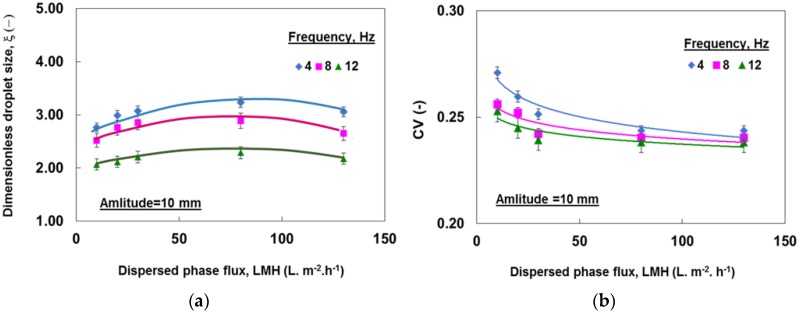
Effect of dispersed phase flux and oscillation frequency on (**a**) droplet size and (**b**) size distribution.

The effect of dispersed phase flux on droplets size and distribution is shown in Figure 7. As can be seen, increasing the dispersed phase flux resulted in an initial increase in droplet size, and a decrease in the coefficient of variations, which is similar to previous observations using high porosity micro-sieve [26].

Such an effect can be attributed to the interaction of several factors. First is the membrane pore activation since only a fraction of the membrane pores are active in the emulsification process. This fraction increases when the dispersed phase applied pressure increases from zero at no flow to ~50% or more depending on the surface porosity and system characteristics. The activation process occurs randomly such that, as certain pores become active, others may stop [27,28]. This behavior could not only result in changes to flow patterns, but also increase the probability for the drops forming at adjacent pores to touch each other. If the surfactant is fast to act, the drops will not coalesce, but rather elongate. This creates another force that has been referred to as the “push-off” force, which further assists droplet detachment against the interfacial tension holding force [29]. For surfaces with close pore spacing, this force will likely play a nontrivial role that could lead to the formation of smaller and more uniform droplet size. This would support, to some extent, the observed change in droplet size at a highly dispersed phase flux, particularly at lower frequencies where droplet size is large in comparison to higher frequencies. It would also partially support the lower CV values observed at flows similar to that reported in previous investigations [3]. In their work, the authors showed that, for small pore spacing membrane and at low shear, the push-off force facilitated the formation of consistent drops at the membrane surface with significantly reduced CV values and limited the otherwise to be expected increase in drop size with increasing injection rate. Their results showed a decrease in droplet size at low shear when the dispersed phase flux was increased. At higher shear, the drop size initially increased, but, after reaching a maximum, it decreased when the flux was increased due to the push-off occurring.

Dispersed phase flow could also affect interfacial tension force and droplet size, which has been reported by previous investigators [22,30]. At higher flow rates, droplets grow at a faster rate, which in turn leads to higher interfacial tension due to a faster surfactant depletion rate at the droplets interface. This results in increasing the interfacial tension holding force, which increases the droplets size. Such an effect, however, would be expected to decrease by either selecting a higher diffusivity surfactant or increasing its concentration and transfer rate, for example, by increasing crossflow velocity (or oscillation in our case). This may partially explain the observed behavior of the decreased effect of the dispersed phase flux on the droplet size at higher oscillation intensities, since the latter results in a “thinning” of the diffusion boundary, which increases the surfactant transfer rate to the forming droplets, thus counteracting the depletion effect caused by droplet growth. A third factor that is also related to the dispersed phase flow is the droplet detachment process, which is not infinitely fast, but requires a certain amount of time. During that time, the droplet volume increases beyond the critical value determined by a force balance and is almost proportional to the dispersed phase injection rate [31]. Further investigation of this phenomenon showed that the detachment time decreases when the continuous phase velocity is increased in crossflow membrane emulsification [32]. In this investigation, surface oscillation provided the equivalent detachment force to that of the continuous phase velocity in crossflow membrane emulsification.

### 3.3. Drop Size Modeling

If the shape of the droplet can be approximated by a sphere before detachment, neglecting forces such as static, lift, and buoyancy forces, the droplet diameter, may be estimated from a torque balance similar to that proposed by a previous investigation, in which the moment of the forces acting towards the pore is balanced by those due to detachment forces [33]. For an oscillatory shear stress τ given by Equation (2), the drag force *F_d_* acting on a droplet with diameter *d* may be approximated by

(7) $$F_{d}=\frac{3}{2}k_{s}\pi \tau d^{2}$$

in which *k*_s_ is a wall correction factor of ~1.7 for a sphere moving parallel to a solid wall in the simple shear flow [34]. For a system interfacial tension γ and a membrane pore diameter *d_p_*, the capillary holding force *F_s_* is

(8) $$F_{s}=\pi d_{p}\gamma$$

A torque balance between the drag and interfacial tension forces may then be written as

(9) $$F_{d}\sqrt{\;{\left(\frac{d}{2}\right)}^{2}-{\left(\frac{d_{p}}{2}\right)}^{2}}=F_{s}\left(\frac{d_{p}}{2}\right)$$

Substituting for drag and interfacial tension forces gives the following expression for the dimensionless droplet size $\xi =d/d_{p}$:

(10) $$\xi =\frac{d}{d_{p}}=\sqrt[{3}]{\frac{2\gamma }{3k_{s}\tau d_{p}}}$$

**Figure 8:**
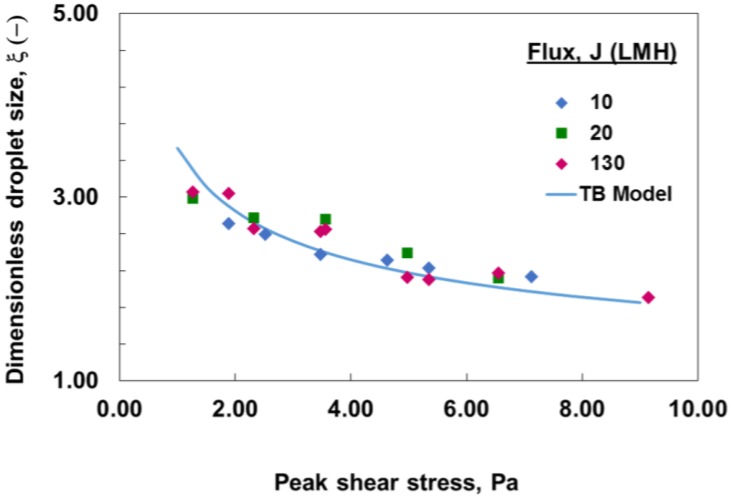
Change in droplet size with oscillatory shear stress and different dispersed phase flux (*J*) for surfactant-stabilized emulsions. Comparison between measured (markers) and torque balance (TB) model predictions (solid line).

Figure 8 shows the comparison between the measured droplet size and those predicted by a simple torque balance model for surfactant-stabilized emulsions, which indicate a reasonable agreement. The deviations shown are most likely due to the fact that simple torque balance modeling does not take into consideration the effect of a dispersed phase flux. It can also be attributed to the possible process of droplets breakage and coalescence, which cannot be predicted by torque balance modeling, and a different analysis approach should be applied in this case.

**Figure 9:**
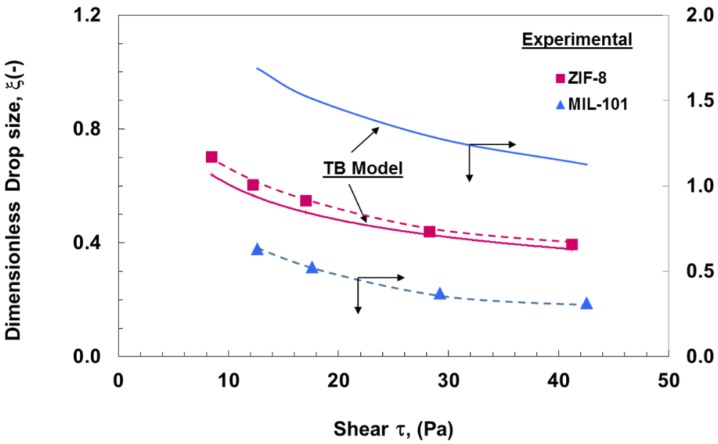
Comparison between measured droplet size (broken lines with markers) and torque balance (TB) model prediction (solid lines) for emulsions stabilized using MOF particles.

For particle-stabilized emulsions, however, and although there was qualitative agreement with the model in terms of droplet size decrease with the shear, there was a significant difference between the measured and predicted droplet sizes for some cases, depending on the hydrophobic or hydrophilic nature of the stabilizing particles, as can be seen in Figure 9. Such observations may suggest that the mechanisms affecting the final droplet size in one case may be different from those at work in the other. A possible hypothesis could be that the strong attachment of the more hydrophobic ZIF-8 particles and its effect on interfacial tension could have likely resulted in the formation of smaller and more stable droplets that resisted breakage. MIL-101 particles, on the other hand, being more hydrophilic, will have less affinity to the oil phase interface and lower surface coverage. The latter may have also been further affected by a possible lower diffusion rate, which, as shown in a recent study, would affect the droplet interfacial characteristics and consequently promote the formation of larger droplets with a greater tendency for breakage to smaller satellite droplets, particularly at higher oscillation intensities [35]. Similar results have been reported when using high shear rates [3].

## 4. Conclusions

Using a commercially available low-cost woven metal microscreen (WMMS)*,* the application of oscillations was found to be effective in producing both surfactant- and particle-stabilized emulsions with reasonably uniform droplet size. For the range of operating conditions investigated, the droplet size and its distribution decreased when both the oscillation frequency and amplitude were increased. Increasing the dispersed phase flow resulted in an increase in droplet size, and the effect became weaker at higher fluxes and as the oscillatory shear increased. For surfactant-stabilized emulsions, using bi-surfactants in both the continuous and dispersed phases resulted in smaller droplet size due to lower interfacial tension. For particle-stabilized emulsions, both the hydrodynamics of the system and the hydrophobic and hydrophilic nature of the stabilizing particles influenced the interfacial properties at the oil–water interface, which in turn affected the final droplet size and distribution with potential droplet breakage. In absence of the latter, and although the simple torque balance model can be used to reasonably predict the average emulsion droplet size, the effect of dispersed phase flow on the surface phenomena and the interaction with the fluid dynamics can result in deviation from the model predictions.

## Abbreviations

The following abbreviations are used in this manuscript:

CV: coefficient of variation
DME: dynamic membrane emulsification
LMH: liter per meter square per hour
MOFs: metal organic frameworks
PE: Pickering emulsions
TB: torque balance
WMMS: woven metal microscreen
